# Supplementary material for: Characterization and Function Analysis of miRNA Editing during Fat Deposition in Chinese Indigenous Ningxiang Pigs
Source: Vet Sci. 2024 Apr 22;11(4):183. doi: 10.3390/vetsci11040183 (PMC11054885; doi:10.3390/vetsci11040183)
Supplement: Supplementary file 1 [file vetsci-11-00183-s001.zip › Figure S1.pdf]

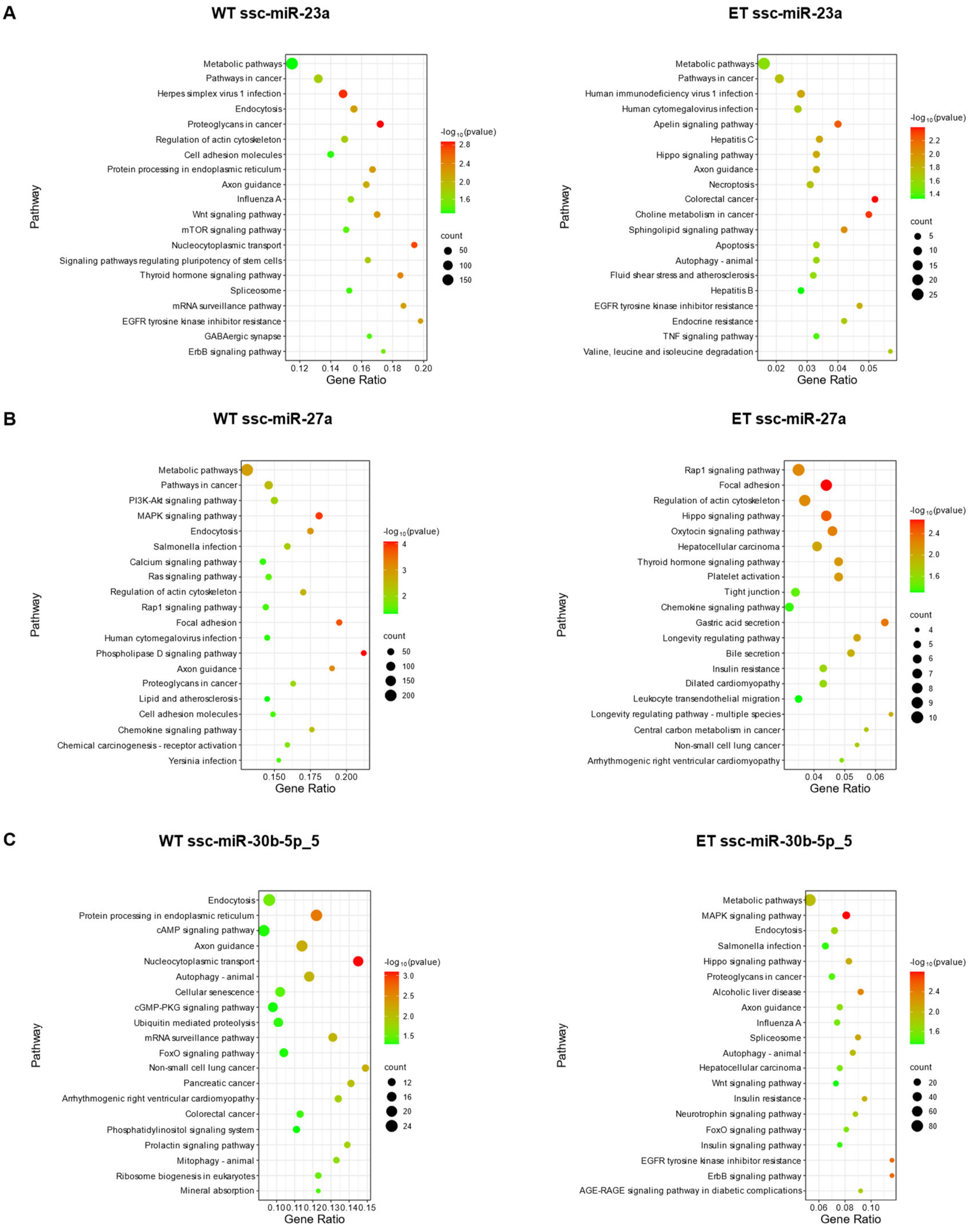
A
WT ssc-miR-23a
Metabolic pathways
Pathways in cancer
Herpes simplex virus 1 infection
Endocytosis
Proteoglycans in cancer
Regulation of actin cytoskeleton
Cell adhesion molecules
Protein processing in endoplasmic reticulum
Axon guidance
Influenza A
Wnt signaling pathway
mTOR signaling pathway
Nucleocytoplasmic transport
Signaling pathways regulating pluripotency of stem cells
Thyroid hormone signaling pathway
Spliceosome
mRNA surveillance pathway
EGFR tyrosine kinase inhibitor resistance
GABAergic synapse
ErbB signaling pathway
Pathway
Gene Ratio
0.12 0.14 0.16 0.18 0.20
-log10(pvalue)
2.8 2.4 2.0 1.6
count
50 100 150
ET ssc-miR-23a
Metabolic pathways
Pathways in cancer
Human immunodeficiency virus 1 infection
Human cytomegalovirus infection
Apelin signaling pathway
Hepatitis C
Hippo signaling pathway
Axon guidance
Necroptosis
Colorectal cancer
Choline metabolism in cancer
Sphingolipid signaling pathway
Apoptosis
Autophagy - animal
Fluid shear stress and atherosclerosis
Hepatitis B
EGFR tyrosine kinase inhibitor resistance
Endocrine resistance
TNF signaling pathway
Valine, leucine and isoleucine degradation
Pathway
Gene Ratio
0.02 0.03 0.04 0.05
-log10(pvalue)
2.2 2.0 1.8 1.6 1.4
count
5 10 15 20 25
B
WT ssc-miR-27a
Metabolic pathways
Pathways in cancer
PI3K-Akt signaling pathway
MAPK signaling pathway
Endocytosis
Salmonella infection
Calcium signaling pathway
Ras signaling pathway
Regulation of actin cytoskeleton
Rap1 signaling pathway
Focal adhesion
Human cytomegalovirus infection
Phospholipase D signaling pathway
Axon guidance
Proteoglycans in cancer
Lipid and atherosclerosis
Cell adhesion molecules
Chemokine signaling pathway
Chemical carcinogenesis - receptor activation
Yersinia infection
Pathway
Gene Ratio
0.150 0.175 0.200
-log10(pvalue)
4 3 2
count
50 100 150 200
ET ssc-miR-27a
Rap1 signaling pathway
Focal adhesion
Regulation of actin cytoskeleton
Hippo signaling pathway
Oxytocin signaling pathway
Hepatocellular carcinoma
Thyroid hormone signaling pathway
Platelet activation
Tight junction
Chemokine signaling pathway
Gastric acid secretion
Longevity regulating pathway
Bile secretion
Insulin resistance
Dilated cardiomyopathy
Leukocyte transendothelial migration
Longevity regulating pathway - multiple species
Central carbon metabolism in cancer
Non-small cell lung cancer
Arrhythmogenic right ventricular cardiomyopathy
Pathway
Gene Ratio
0.04 0.05 0.06
-log10(pvalue)
2.4 2.0 1.6
count
4 5 6 7 8 9 10
C
WT ssc-miR-30b-5p_5
Endocytosis
Protein processing in endoplasmic reticulum
cAMP signaling pathway
Axon guidance
Nucleocytoplasmic transport
Autophagy - animal
Cellular senescence
cGMP-PKG signaling pathway
Ubiquitin mediated proteolysis
mRNA surveillance pathway
FoxO signaling pathway
Non-small cell lung cancer
Pancreatic cancer
Arrhythmogenic right ventricular cardiomyopathy
Colorectal cancer
Phosphatidylinositol signaling system
Prolactin signaling pathway
Mitophagy - animal
Ribosome biogenesis in eukaryotes
Mineral absorption
Pathway
Gene Ratio
0.10 0.11 0.12 0.13 0.14 0.15
-log10(pvalue)
3.0 2.5 2.0 1.5
count
12 16 20 24
ET ssc-miR-30b-5p_5
Metabolic pathways
MAPK signaling pathway
Endocytosis
Salmonella infection
Hippo signaling pathway
Proteoglycans in cancer
Alcoholic liver disease
Axon guidance
Influenza A
Spliceosome
Autophagy - animal
Hepatocellular carcinoma
Wnt signaling pathway
Insulin resistance
Neurotrophin signaling pathway
FoxO signaling pathway
Insulin signaling pathway
EGFR tyrosine kinase inhibitor resistance
ErbB signaling pathway
AGE-RAGE signaling pathway in diabetic complications
Pathway
Gene Ratio
0.06 0.08 0.10
-log10(pvalue)
2.4 2.0 1.6
count
20 40 60 80

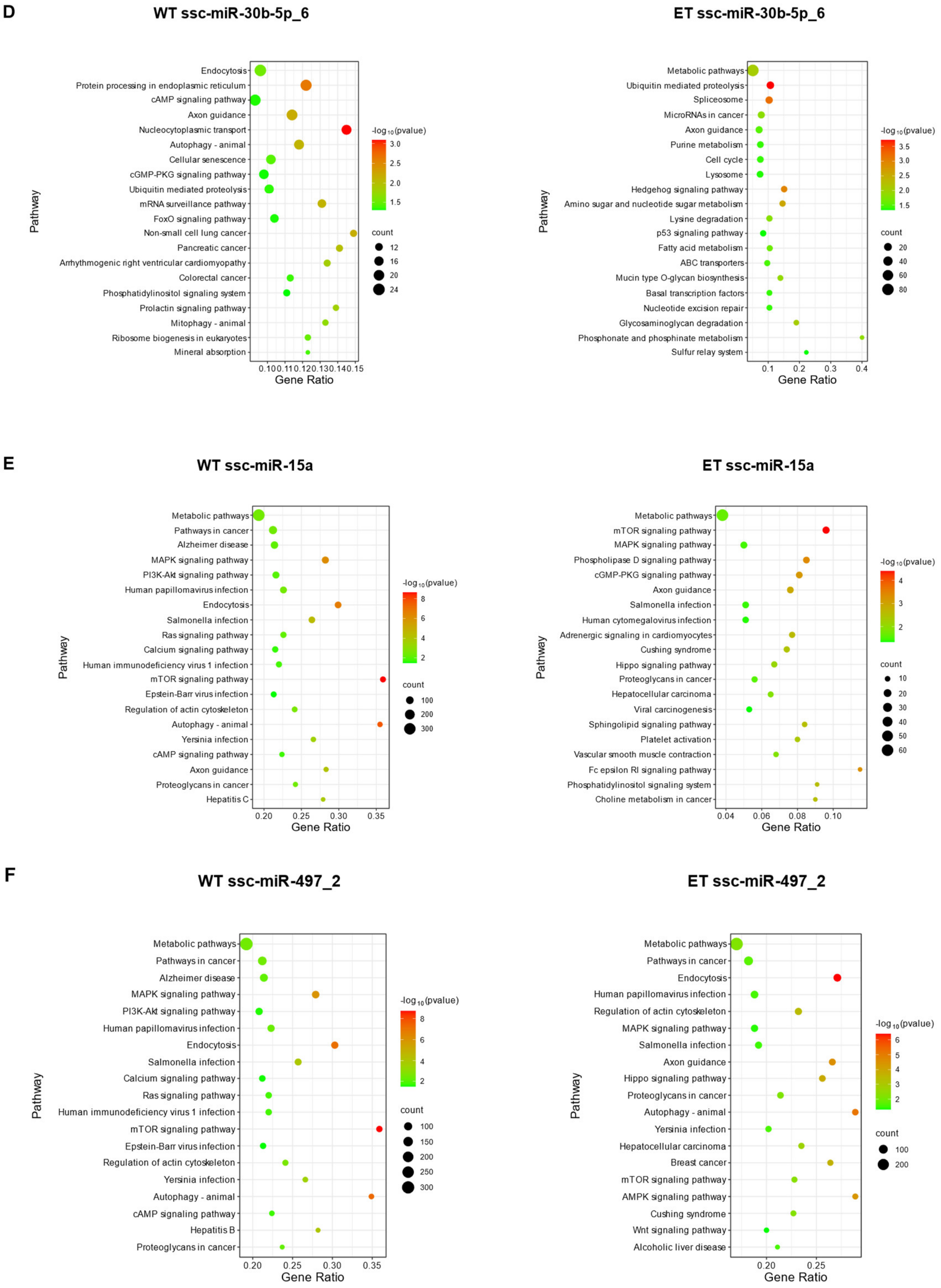

D
WT ssc-miR-30b-5p_6
Endocytosis
Protein processing in endoplasmic reticulum
cAMP signaling pathway
Axon guidance
Nucleocytoplasmic transport
Autophagy - animal
Cellular senescence
cGMP-PKG signaling pathway
Ubiquitin mediated proteolysis
mRNA surveillance pathway
FoxO signaling pathway
Non-small cell lung cancer
Pancreatic cancer
Arrhythmogenic right ventricular cardiomyopathy
Colorectal cancer
Phosphatidylinositol signaling system
Prolactin signaling pathway
Mitophagy - animal
Ribosome biogenesis in eukaryotes
Mineral absorption
Pathway
Gene Ratio
0.10 0.11 0.12 0.13 0.14 0.15
-log10(pvalue)
3.0 2.5 2.0 1.5
count
12 16 20 24
ET ssc-miR-30b-5p_6
Metabolic pathways
Ubiquitin mediated proteolysis
Spliceosome
MicroRNAs in cancer
Axon guidance
Purine metabolism
Cell cycle
Lysosome
Hedgehog signaling pathway
Amino sugar and nucleotide sugar metabolism
Lysine degradation
p53 signaling pathway
Fatty acid metabolism
ABC transporters
Mucin type O-glycan biosynthesis
Basal transcription factors
Nucleotide excision repair
Glycosaminoglycan degradation
Phosphonate and phosphinate metabolism
Sulfur relay system
Pathway
Gene Ratio
0.1 0.2 0.3 0.4
-log10(pvalue)
3.5 3.0 2.5 2.0 1.5
count
20 40 60 80
E
WT ssc-miR-15a
Metabolic pathways
Pathways in cancer
Alzheimer disease
MAPK signaling pathway
PI3K-Akt signaling pathway
Human papillomavirus infection
Endocytosis
Salmonella infection
Ras signaling pathway
Calcium signaling pathway
Human immunodeficiency virus 1 infection
mTOR signaling pathway
Epstein-Barr virus infection
Regulation of actin cytoskeleton
Autophagy - animal
Yersinia infection
cAMP signaling pathway
Axon guidance
Proteoglycans in cancer
Hepatitis C
Pathway
Gene Ratio
0.20 0.25 0.30 0.35
-log10(pvalue)
8 6 4 2
count
100 200 300
ET ssc-miR-15a
Metabolic pathways
mTOR signaling pathway
MAPK signaling pathway
Phospholipase D signaling pathway
cGMP-PKG signaling pathway
Axon guidance
Salmonella infection
Human cytomegalovirus infection
Adrenergic signaling in cardiomyocytes
Cushing syndrome
Hippo signaling pathway
Proteoglycans in cancer
Hepatocellular carcinoma
Viral carcinogenesis
Sphingolipid signaling pathway
Platelet activation
Vascular smooth muscle contraction
Fc epsilon RI signaling pathway
Phosphatidylinositol signaling system
Choline metabolism in cancer
Pathway
Gene Ratio
0.04 0.06 0.08 0.10
-log10(pvalue)
4 3 2
count
10 20 30 40 50 60
F
WT ssc-miR-497_2
Metabolic pathways
Pathways in cancer
Alzheimer disease
MAPK signaling pathway
PI3K-Akt signaling pathway
Human papillomavirus infection
Endocytosis
Salmonella infection
Calcium signaling pathway
Ras signaling pathway
Human immunodeficiency virus 1 infection
mTOR signaling pathway
Epstein-Barr virus infection
Regulation of actin cytoskeleton
Yersinia infection
Autophagy - animal
cAMP signaling pathway
Hepatitis B
Proteoglycans in cancer
Pathway
Gene Ratio
0.20 0.25 0.30 0.35
-log10(pvalue)
8 6 4 2
count
100 150 200 250 300
ET ssc-miR-497_2
Metabolic pathways
Pathways in cancer
Endocytosis
Human papillomavirus infection
Regulation of actin cytoskeleton
MAPK signaling pathway
Salmonella infection
Axon guidance
Hippo signaling pathway
Proteoglycans in cancer
Autophagy - animal
Yersinia infection
Hepatocellular carcinoma
Breast cancer
mTOR signaling pathway
AMPK signaling pathway
Cushing syndrome
Wnt signaling pathway
Alcoholic liver disease
Pathway
Gene Ratio
0.20 0.25
-log10(pvalue)
6 5 4 3 2
count
100 200

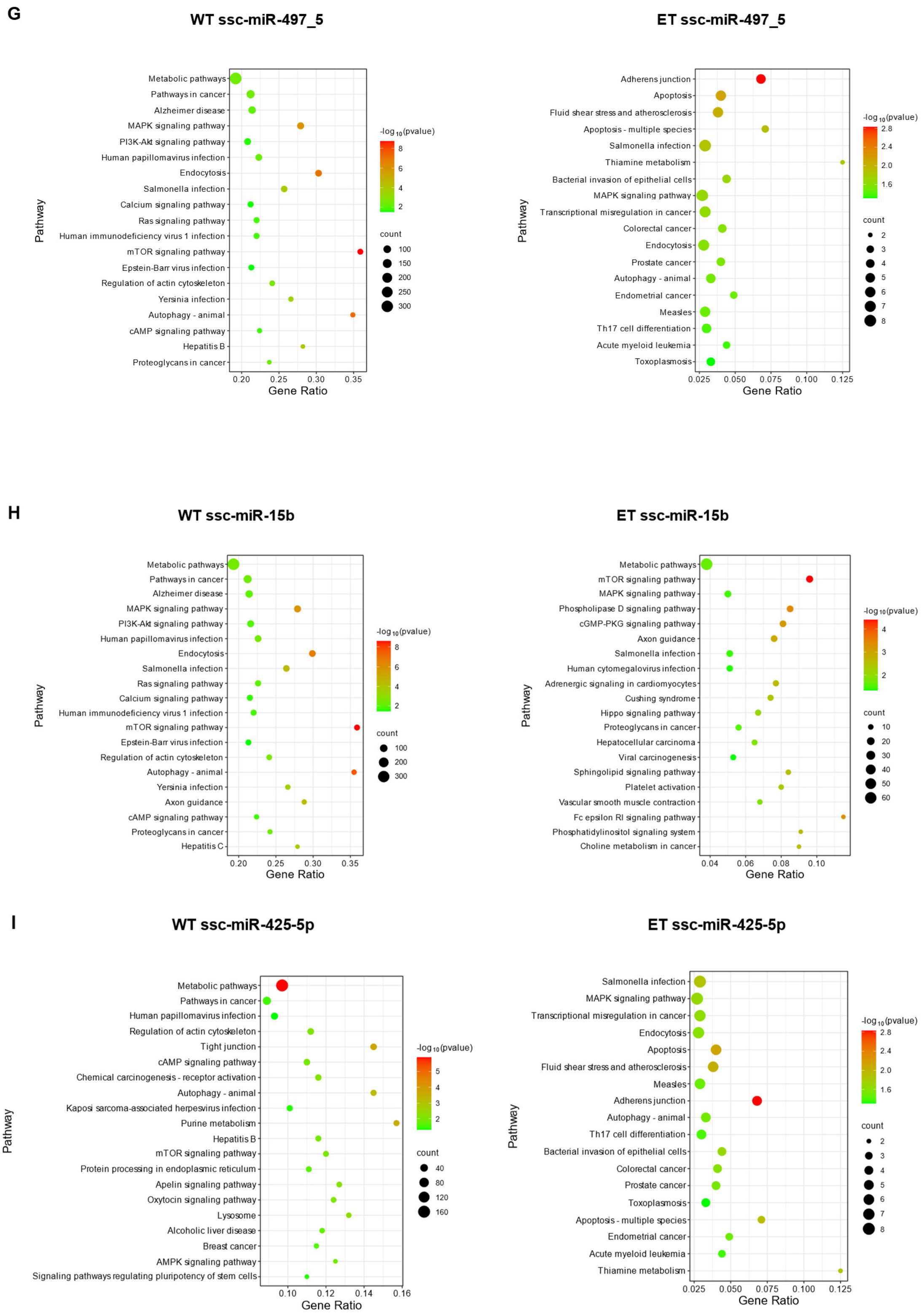

Figure S1: KEGG enrichment analysis results of WT and ET miRNAs target genes. ssc-miR-23a (A), ssc-miR-27a (B), ssc-miR-30b-5p_5 (C), ssc-miR-30b-5p_6 (D), ssc-miR-15a (E), ssc-miR-497_2 (F); ssc-miR-497_5 (G) , ssc-miR-15b (H), ssc-miR-425-5p (I).
